# Quantification of fibrous cap thickness in intracoronary optical coherence tomography with a contour segmentation method based on dynamic programming

**DOI:** 10.1007/s11548-015-1164-7

**Published:** 2015-03-05

**Authors:** Guillaume Zahnd, Antonios Karanasos, Gijs van Soest, Evelyn Regar, Wiro Niessen, Frank Gijsen, Theo van Walsum

**Affiliations:** 1 Biomedical Imaging Group Rotterdam, Departments of Radiology and Medical Informatics, Erasmus Medical Center, P.O. Box 2040, 3000 CA Rotterdam, The Netherlands; 2 Department of Interventional Cardiology, Thorax Center, Erasmus MC, Rotterdam, The Netherlands; 3 Department of Biomedical Engineering, Thorax Center, Erasmus MC, Rotterdam, The Netherlands

**Keywords:** Coronary artery, Optical coherence tomography, Interventional imaging, Thin-cap fibroatheroma, Contour segmentation, Dynamic programming, Preoperative planning

## Abstract

**Objectives:**

Fibrous cap thickness is the most critical component of plaque stability. Therefore, in vivo quantification of cap thickness could yield valuable information for estimating the risk of plaque rupture. In the context of preoperative planning and perioperative decision making, intracoronary optical coherence tomography imaging can provide a very detailed characterization of the arterial wall structure. However, visual interpretation of the images is laborious, subject to variability, and therefore not always sufficiently reliable for immediate decision of treatment.

**Methods:**

A novel semiautomatic segmentation method to quantify coronary fibrous cap thickness in optical coherence tomography is introduced. To cope with the most challenging issue when estimating cap thickness (namely the diffuse appearance of the anatomical abluminal interface to be detected), the proposed method is based on a robust dynamic programming framework using a geometrical a priori. To determine the optimal parameter settings, a training phase was conducted on 10 patients.

**Results:**

Validated on a dataset of 179 images from 21 patients, the present framework could successfully extract the fibrous cap contours. When assessing minimal cap thickness, segmentation results from the proposed method were in good agreement with the reference tracings performed by a medical expert (mean absolute error and standard deviation of $22\,\pm \,18\,\upmu \hbox {m},\hbox { R}\,=\,.73$) and were similar to inter-observer reproducibility ($21\,\pm \,19\,\upmu \hbox {m}$, R = .74), while being significantly faster and fully reproducible.

**Conclusion:**

The proposed framework demonstrated promising performances and could potentially be used for online identification of high-risk plaques.

## Introduction

**Fig. 1:**
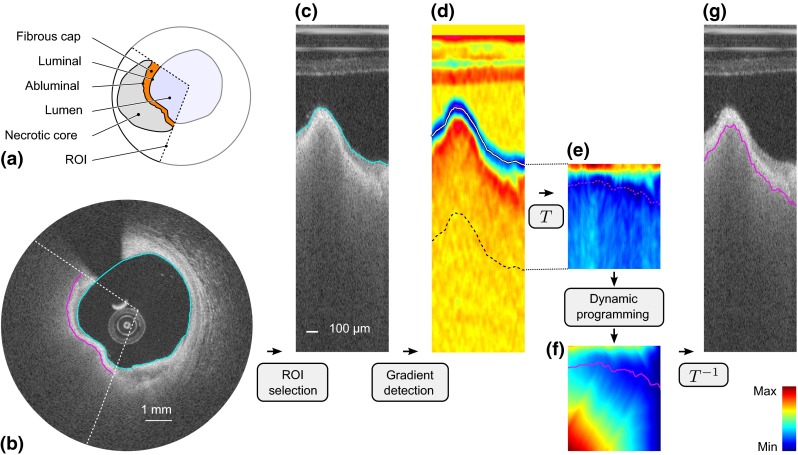
Segmentation framework. **a** Cartoon depicting the region of interest (ROI, *dashed lines*) encompassing the fibrous cap. **b** OCT image of an in vivo human coronary artery, in Cartesian coordinates, with the resulting luminal (*cyan line*) and abluminal (*magenta line*) segmentation contours. **c** ROI in polar coordinates, with the luminal contour (*cyan line*). **d** Gradient image $I_G$. **e** Transformed cost image ${\mathcal {C}}_T$. **f** Cumulated cost ${\mathbb {C}}$, with the optimal path (*magenta line*). **g** Resulting abluminal segmentation contour

Coronary artery disease is the most common cause of human mortality and morbidity in industrialized countries. Acute coronary syndrome (ACS), the most severe manifestation of atherosclerotic disease, is principally caused by acute coronary thrombosis, which is mainly provoked by plaque rupture [17]. The morphological characteristics of such plaques that are prone to rupture (also dubbed “high-risk” or “vulnerable” plaques) are (1) a large lipid necrotic core, (2) an overlying thin fibrous cap, and (3) dense macrophage infiltration (Fig. 1a) [4]. These plaques are also known as thin-cap fibroatheromas (TCFAs) and are considered the precursor phenotype of plaque rupture. The most critical component of plaque stability is fibrous cap thickness, i.e., thinner caps being more prone to rupture than thicker caps, and the threshold of $65\,\upmu \hbox {m}$ has been widely adopted to identify high-risk lesions [11]. Accordingly, identification of vulnerable plaques could potentially guide appropriate surgical treatments such as percutaneous coronary intervention (e.g., balloon angioplasty or stent placement) prior to the occurrence of an event. Therefore, in vivo quantification of fibrous cap thickness represents a major clinical challenge.

Intravascular optical coherence tomography (OCT) is a catheter-based imaging modality that enables tissues to be visualized in vivo at a near-histology resolution (10–20 $\upmu \hbox {m})$ and in a minimally invasive way [3]. In a similar fashion as intravascular ultrasound, the inner circumference of the vessel is investigated by the probe spinning along its axis while being pulled back. At each angular step, a so-called A-line signal is acquired via the emission and reception of near-infrared light (center wavelength of 1280–1350 nm). A stack of consecutive cross-sectional images along the length of the assessed artery segment is then reconstructed by converting the intensity and echo time of all A-lines into a gray-scale representation (Fig. 1b). The very high spatial resolution of OCT enables an accurate characterization of the structure of the most superficial layers of the arterial wall and can indicate the degree of subclinical atherosclerotic lesion formation [14]. Moreover, OCT is currently the only in vivo imaging modality with which fibrous cap thickness, the most critical component of plaque stability, can be assessed accurately [10]. Therefore, OCT can potentially be used for in vivo identification of high-risk plaques.

Although OCT images are acquired online during intervention, fibrous cap thickness quantification is currently performed manually offline [2, 14]. The two major drawbacks that hinder such manual image analysis are (1) the procedure is cumbersome and time-consuming, and (2) results are subject to a certain degree of variability between different analysts [9, 10]. Moreover, segmentation of the fibrous cap abluminal interface is a challenging task, as fibroatheromas consist of progressively unraveling tissues and are visualized in OCT as signal-poor regions with diffuse contours and high signal attenuation (Fig. 1a,b) [14]. Therefore, the clinical need of immediate and reliable information is not fully met by current procedures based on manual image analysis.

Aiming to provide reliable and quantified information during OCT analysis in the intervention room, various (semi)automated computerized methods have recently been proposed. The attenuation coefficient of the backscattered light has been used in several classification-based approaches [12, 16, 20]. These methods were successfully used to identify and locate different types of tissues (i.e., healthy wall sections, lipid, calcific and fibrous tissues). Nevertheless, such techniques are not devised to provide information regarding the actual delineation of anatomical interfaces and could not be used to assess fibrous cap thickness. A seminal study was proposed to specifically assess fibrous cap thickness [19]. This method, based on contour segmentation by means of dynamic programming, was applied to extract both luminal and abluminal interfaces of the fibroatheromas and could quantify cap thickness. However, since this method did not exploit geometrical a priori features, results could have potentially been hindered in images with an eccentric catheter position within the lumen. Another semiautomatic method was introduced to identify the different tissue types and segment the wall layers [5]. In this approach, contour segmentation was based on intensity thresholding. Nevertheless, although the results of this study look promising, fibrous cap thickness was not investigated per se.

The present study aims at introducing and evaluating a framework designed to quantify fibrous cap thickness of fibroatheromas in intracoronary OCT. The principal contribution of this work is a robust contour segmentation method devised to extract the fuzzy abluminal interface of the fibrous cap. This novel framework is based on a dynamic programming approach that previously showed successful results on the common carotid artery wall in B-mode ultrasound [24]. The accuracy of the present method was validated in a set of 179 cross-sectional OCT images acquired in vivo from 21 different patients and demonstrated a similar accuracy compared to the tracings manually performed by two experienced analysts.

## Materials and methods

The present segmentation framework is based on three principal phases, (1) a manual initialization aiming to indicate the presence of the fibrous cap to be analyzed, (2) the automatic extraction of the luminal interface in the objective to localize the wall contour, and (3) the automatic extraction of the abluminal interface, which is subsequently exploited to assess the actual cap thickness. An overview of the method is presented in Fig. 1. The outline of this section is the following. First, we introduce a contour segmentation scheme based on dynamic programming, which is exploited in the phases (2) and (3) of our framework. Then, we detail the three principal phases of our framework.

### Dynamic programming

Dynamic programming is an efficient method to find the globally optimal solution in combinatory analysis [1]. In the present context, contour segmentation is performed in the polar domain. Given an image $I$, the anatomical interface to be extracted corresponds to a curve running from the left to the right border of the image, as depicted in Fig. 1c. We thus address the issue of determining, among all the potential candidate contours, the one that best describes the actual (1) location and (2) shape (i.e., smoothness) of the anatomical interface. Toward this objective, we propose a specific implementation of a dynamic programming framework based on front propagation [6].

#### Cost function

Since the anatomical interfaces to be extracted are located on regions of the image showing a strong intensity transition, the first step consists in locally enhancing the vertical intensity gradient of the image. One should notice that this transition is positive for the luminal interface (i.e., from dark lumen to bright tissues) and negative for the abluminal interface (i.e., from bright fibrous tissues to dark lipid pool), as depicted in Fig. 1c,d. The gradient image $I_G$ is then built according to:

1 $$\begin{aligned} I_G = \pm G'*I, \end{aligned}$$

with $(*)$ the convolution operator and $G'$ the first derivative of a Gaussian function of standard deviation $\sigma$. The $\pm$ sign corresponds to the gradient orientation and is determined according to the processed interface, namely it is positive for the luminal contour and negative for the abluminal contour. Finally, a cost function ${\mathcal {C}}$ is built such as:

2 $$\begin{aligned} {\mathcal {C}}={\mathcal {N}}_{[0,1]}(-I_G), \end{aligned}$$

with ${\mathcal {N}}_{[0,1]}$ representing the normalization of a set of values to the positive interval $[0,1]$ (viz.: the set is first linearly scaled in such way that the minimum value becomes equal to zero, and then the set is divided by the maximum value). In this image ${\mathcal {C}}$, the points most likely to represent the location of the analyzed interface correspond to the points with the lowest cost (Fig. 1e).

#### Front propagation

**Fig. 2:**
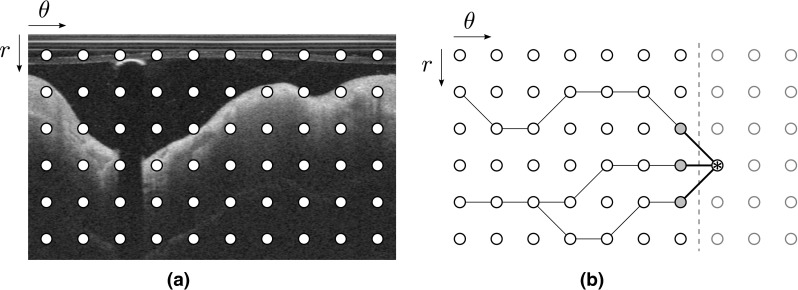
Schematic representation of the front propagation scheme, corresponding to the panel (f) in Fig. 1. **a** Original polar image, with the pixels represented by the *nodes* (in this example, the layout is coarse for improved visibility). **b** Left-to-right front propagation, with the current location of the front indicated by the *vertical dashed line*. The current node is indicated by an *asterisk*, and the connected gray nodes correspond to the set of potential neighbors. In this example, the number 2*N* + 1 of horizontally reachable neighbors is equal to 3. The *black lines* connecting the nodes represent the successive backtracking steps from a given node to the left border of the image. Please note that in the case of segmenting the luminal interface, the nodes of the upper row correspond to the top of the polar image (as shown in this example), whereas in the case of the segmenting the abluminal interface, the nodes of the upper row correspond to the luminal interface

We now present a dynamic programming strategy to determine the path that runs in the cost image ${\mathcal {C}}$ from left-to-right with the minimum cumulated cost ${\mathbb {C}}$. A schematic representation of this front propagation approach is displayed in Fig. 2. The proposed approach extends a previously proposed method [23, 24] and takes into account both the image feature (i.e., strong intensity gradient locally corresponding to a low cost in ${\mathcal {C}}$) and a geometrical constraint (i.e., the shape a priori that describes a smooth structure). Therefore, high cost values as well as vertical displacement are penalized when generating the cumulated cost function ${\mathbb {C}}$, as detailed in Eq. 3 (Fig. 1f).

3 $$\begin{aligned} {\mathbb {C}}(r, \theta +1)= & {} \min _{d_r \in \{ -N,\dots 0,\dots N \}} \Big \{ {\mathbb {C}}(r+d_r, \theta ) + \left( {\mathcal {C}}(r,\theta +1)\right. \nonumber \\&\left. +\, {\mathcal {C}}(r+d_r,\theta ) \right) \cdot \left( 1+ \alpha \cdot d_r^{\beta } \right) \Big \}, \end{aligned}$$

with $(r,\theta )$ the vertical and horizontal coordinates, $d_r$ the vertical displacement of the path between two consecutive points, and 2*N* + 1 the number of reachable neighbors. The smoothness of the path is ruled by the positive parameters $\alpha$ and $\beta$. More specifically, the overall flexibility of the path is controlled by $\alpha$ (i.e., small $\alpha$ values enable vertical transitions of the path, and large $\alpha$ values favor long horizontal plateaus), and the roughness of the path is controlled by $\beta$ (i.e., small $\beta$ values yield contours that are locally spiky, and large $\beta$ values impose smooth contours).

Prior to computation, the left column of ${\mathbb {C}}$ (i.e., $\theta \,=\,1$) is initialized to zero: in this implementation, each node on the left border acts as a seed and is a potential starting point for the final optimal path. It is also noteworthy that the weight of each candidate link depends on the cost of the edge connecting the currently evaluated node with the candidate node (viz.: ${\mathcal {C}}(r,\theta +1) + {\mathcal {C}}(r+d_r,\theta )$ in Eq. 3), rather than the cost of the current node alone (viz.: ${\mathcal {C}}(r,\theta +1)$). Therefore, this implementation is independent of the direction (i.e., left-to-right or right-to-left) of the front propagation.

#### Back tracking

In the objective to extract the globally optimal path, a backtracking scheme is adopted. Since ${\mathbb {C}}$ is constructed using a penalty that depends of the vertical distance between the nodes (Eq. 3), a classical gradient descent in ${\mathbb {C}}$ cannot be performed to extract the minimal cost path. Instead, during the previously described propagation of the front, for each node ${\mathcal {C}}(r,\theta +1)$, neighboring information is memorized by storing the vertical coordinate $r+dr$ of the best candidate node ${\mathcal {C}}(r+dr,\theta )$, as shown in Fig. 2. Once the cumulated cost function ${\mathbb {C}}$ is entirely built, the ending point of the path is determined by the node with the minimal cumulated cost located on the right border of the image. Finally, the total path is extracted via backtracking by iteratively connecting the nodes using the stored neighboring information, from the right to the left border of the image.

### Initialization and preprocessing

The present framework starts with the user manually performing a quick and simple initialization phase. For a given pullback, this operation consists in (1) visually detecting the presence of a necrotic core covered by a fibrous cap and (2) manually indicating the region of interest (ROI) to be analyzed. The ROI was defined by an arc encompassing the fibrous cap, as displayed in Fig. 1a, b. After this operation has been performed, the region shadowed by the guidewire is easily masked out using an approach similar to the one proposed in [19].

### Lumen segmentation

The luminal interface is represented by a positive intensity transition (i.e., from dark lumen to bright tissues) and is generally well perceptible. The luminal contour is easily extracted by applying the previously described dynamic programming approach to the image $I$.

### Abluminal interface segmentation

The abluminal interface is represented by a negative intensity transition (i.e., from bright fibrous tissues to dark lipid pool) and is generally more diffuse and fuzzy. Prior to applying the dynamic programming segmentation method, the ROI manually selected by the user is extracted from the image $I$, as depicted in Fig. 1c. Then, a spatial transformation $T$ is applied to the ROI. The aim of this transformation $T$ is to generate a sub-image ${\mathcal {C}}_T$ in which the luminal interface corresponds to a straight horizontal line in the polar domain (Fig. 1e). The cost function ${\mathcal {C}}$ is thus shifted line-by-line to match the vertical origin with respect to the luminal contour rather than to the probe location. The rationale of our approach is based on the fact that as the fibrous cap thickness does not undergo large variations within adjacent sites, we can exploit a geometrical a priori to cope with the diffuse appearance of the anatomical interface. In the transformed sub-image ${\mathcal {C}}_T$, the abluminal contour that needs to be extracted is henceforth expected to correspond to a nearly horizontal structure. Subsequently, the dynamic programming segmentation method is applied to ${\mathcal {C}}_T$. Finally, the actual location of the abluminal interface in the original image is determined by applying the corresponding inverse spatial transformation $T^{-1}$ onto the extracted optimal path (Fig. 1g).

## Experiments

### Data collection and study population

The OCT imaging database of Thoraxenter, Erasmus MC (Rotterdam, The Netherlands), was screened for native coronary artery OCT pullbacks containing fibroatheromas. Fibroatheromas were defined as necrotic core containing regions with the maximum circumferential extent (arc) exceeding one quadrant of the cross section. Thirty one patients (mean age $61.3\,\pm \,8.4~\text {years old}$, 25 males) suffering from coronary artery disease were randomly selected from the database and included in our study. The only inclusion criterion was the presence of fibroatheromas in the acquired pullbacks. Informed consent was acquired from the patients for the use of their imaging data. All procedures followed were in accordance with the ethical standards of the responsible committee on human experimentation (institutional and national) and with the Helsinki Declaration of 1975, as revised in 2008 (5). Pullbacks were acquired in the catheterization laboratory of Erasmus MC for clinical indications, using the C7XR frequency-domain system and the Dragonfly intracoronary imaging catheter (Lightlab/St Jude, Minneapolis, MN, USA). Image acquisition was performed with a previously described non-occlusive technique [14]. Briefly, after positioning the OCT catheter distally to the segment of interest, it was pulled back automatically at 20 mm/s with simultaneous contrast infusion through the guiding catheter by a power injector (flush rate 3-4$\hbox { ml/s}$). Images were acquired at the rate of 100 frames/s (corresponding to 54000 A-lines/s), over an average total length of 54 mm along the vessel, resulting in a stack of 271 frames. The central bandwidth of the near-infrared light was 1310 nm, and the spatial resolution of the system was 20 and 30 $\upmu \hbox {m}$ in the axial and lateral directions, respectively. The depth of the scan range was 4.3 mm, and acquired images were sampled at $504 \times 968$ pixels per frame, with an isotropic pixel size of $4.5~\upmu \mathrm{m}$.

### Image analysis procedure

For each analyzed pullback, an analyst ${\mathcal {A}}_1$ selected a series of consecutive images where a necrotic core with an overlying fibrous cap could be observed visually. Definition of image features identifying a necrotic core was signal-poor regions with diffuse contours and high signal attenuation [14]. Subsequently, ${\mathcal {A}}_1$ indicated, in each selected frame, the limits of the ROI encompassing the fibrous cap (Fig. 1a, b). All that information was stored and subsequently used by the automatic segmentation method, the expert ${\mathcal {A}}_1$, as well as an additional analyst ${\mathcal {A}}_2$ to perform, blinded to the results of others, the extraction of the abluminal interface of the fibrous cap. All tracings realized by the human analysts were performed in the Cartesian domain via an effective graphical interface that was developed in-house for this purpose. The two experts are specialists in vascular imaging and OCT. They received identical instructions and were trained on the new segmentation software during 1 month prior to this study.

### Parameter settings

#### Luminal interface

Segmentation of the luminal interface does not present any particular challenge. The proposed segmentation framework was therefore applied on the entire circumference of all images with the following heuristically determined parameter settings: smoothness parameters, $\alpha =0.1$ and $\beta =1$; standard deviation of the Gaussian filter, $\sigma = 90~\upmu \hbox {m}$; number of reachable neighbors, $2N+1=41$.

#### Abluminal interface

**Fig. 3:**
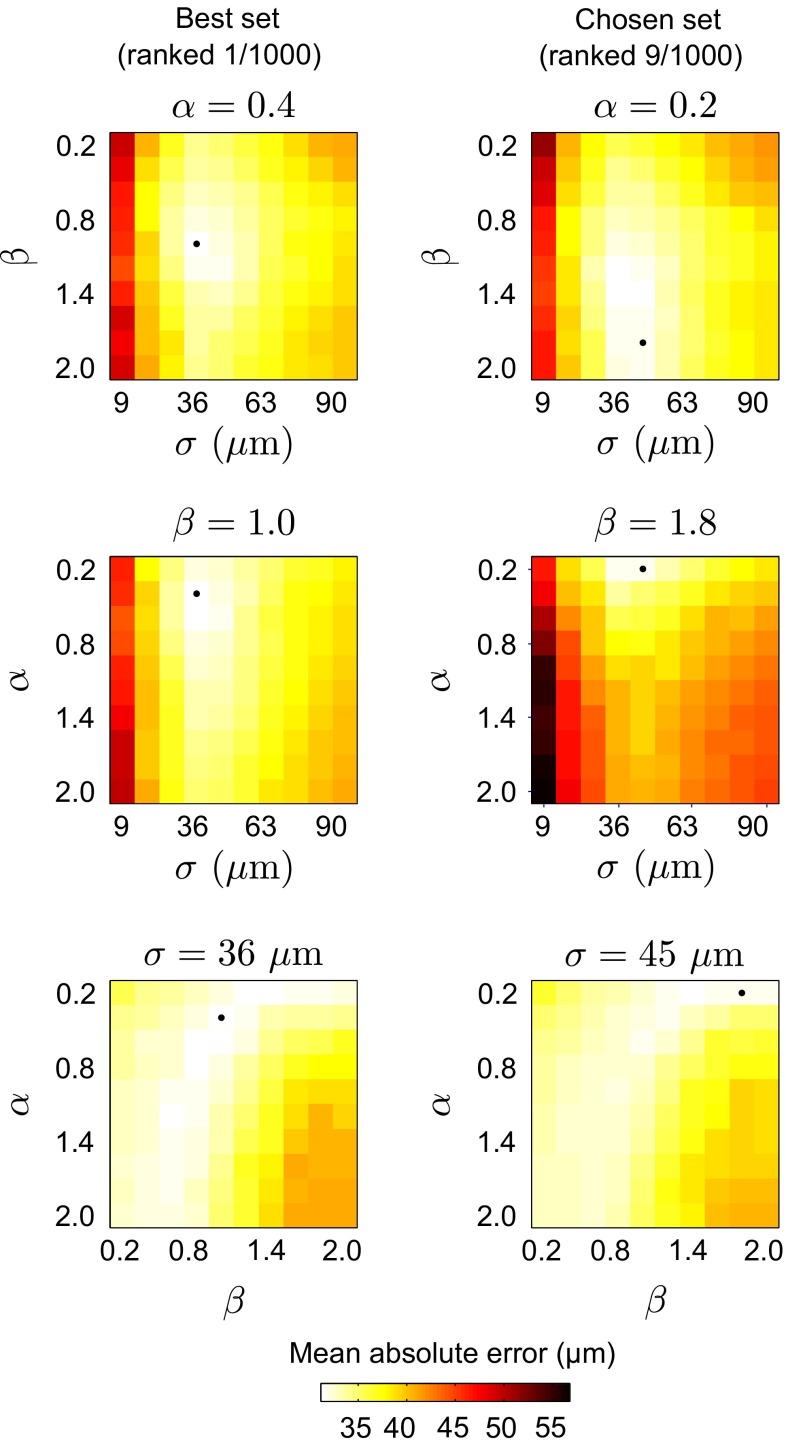
Mean absolute segmentation error of the fibrous cap abluminal interface, between the automatic framework and the manual tracings of the analyst ${\mathcal {A}}_1$, in function of the parameter settings $\{\alpha , \beta , \sigma \}$. In each panel, the location of the minimal error is indicated by the *black dot*

Aiming to accurately extract the abluminal contour of the fibrous cap, the optimal parameter settings were determined by means of a training phase. In this purpose, a training set was generated by randomly selecting a subsample of $\varOmega _1=10$ pullbacks among the cohort of 31 participants. During the training phase, the proposed framework was repeatedly applied to the training set, with 1000 different sets of $\{\alpha ,\beta ,\sigma \}$ parameter settings, as displayed in Table 1. The number $2N+1$ of reachable neighbors was equal to 41 to reduce the search space while still allowing the path to follow the curvature of the analyzed interface. A score was then attributed to each set of parameter settings, by calculating, for every frame of the training set, the mean error between the reference abluminal contour manually traced by ${\mathcal {A}}_1$ and the corresponding segmentation contour resulting from the proposed framework. Finally, the optimal set of $\{\alpha ,\beta ,\sigma \}$ parameter settings was determined by visually inspecting the contours of the 10 best ranked sets and selecting the configuration yielding the contours with the most realistic appearance. The selected configuration was the ninth best ranked set, with a mean absolute error of $32\pm 40~\upmu \text {m}$. The parameters corresponding to the chosen set were as follows: smoothness parameters, $\alpha =0.2$ and $\beta =1.8$; standard deviation of the Gaussian filter, $\sigma = 45~\upmu \text {m}$. For comparison purpose, the mean absolute error corresponded to $31\pm 41~\upmu \text {m}$ for the best ranked set $(\{\alpha ,\beta ,\sigma \}=\{0.4, 1.0, 36~\upmu \text {m}\})$, and to $57\pm 74~\upmu \text {m}$ for the worst ranked set $(\{\alpha ,\beta ,\sigma \}=\{2.0, 2.0, 9~\upmu \text {m}\})$. Moreover, the difference between the error distributions corresponding to the chosen set and the best ranked set yielded a zero bias and a 95 % confidence interval equal to $[-3, 3]~\upmu \text {m}$. By defining a zone of clinical indifference equal to $9~\upmu \text {m}$ (i.e., $\pm 1$ pixel), we can conclude that the accuracy of the chosen set is statistically equivalent to the accuracy of the best ranked set. Resulting errors in function of the $\{\alpha ,\beta ,\sigma \}$ parameter settings are displayed in Fig. 3.

**Table 1:** Values of the parameter settings used during the training phase of the method

Parameter	Number of different values	Min	Max	Increment step
$\alpha$	10	0.2	2.0	0.2
$\beta$	10	0.2	2.0	0.2
$\sigma$	10	$9\,\upmu \hbox {m}$	$90 \,\upmu \hbox {m}$	$9 \,\upmu \hbox {m}$

### Fibrous cap thickness evaluation

The performance of the proposed segmentation framework was evaluated as follow: a testing set was generated with the remaining $\varOmega _2=21$ pullbacks, and then the segmentation framework was applied onto the testing set with the previously determined optimal parameter settings. For each analyzed image, thickness of the fibrous cap was assessed in the Cartesian domain, for our automatic method as well as the two analysts ${\mathcal {A}}_1$ and ${\mathcal {A}}_2$. For a given point of the abluminal interface of the fibrous cap, the measure was performed on the line going through the center of the lumen and the assessed point. Cap thickness corresponded to the distance between the two points defined by the intersection of this line with both luminal and abluminal interfaces. For each image, two different measurements were realized to evaluate cap thickness, namely (i) as a vector describing each A-line of the analyzed ROI, and (ii) as the thinnest portion within the frame.

### Manual correction of the abluminal contour

The robustness of the proposed segmentation method was also evaluated in the training set by the expert ${\mathcal {A}}_2$ visually assessing each resulting segmentation contour of the abluminal interface and manually correcting it if necessary. More precisely, one or several control points were manually placed by ${\mathcal {A}}_2$ to correct the automatic segmentation when the analyst did not agree with the original resulting contour. The corrected segmentation contour was generated by means of a modified implementation of the dynamic programming method detailed in “Dynamic programming”. Immediately after the generation of the cost function ${\mathcal {C}}$, a modified cost function ${\mathcal {C}}'$ was built using the following approach. For each manually defined control point $p(\theta , r)$, the node $(\theta , r)$ of the cost function ${\mathcal {C}}'$ was set to zero, and all other nodes of the column $\theta$ were set to an infinity value (Fig. 2). The following steps of the dynamic programming method were then applied to the cost function ${\mathcal {C}}'$. As a consequence, the resulting contour corresponded to a path going through all the control points while still performing a search in the regions that were not corrected.^1^

## Results

Among the 31 involved patients, the average number of analyzed images per individual pullback was $8.4 \pm 1.7$ (range 5–10) consecutive frames, with a total of 261 analyzed images. The average length of the analyzed arc per image was $30\pm 16\,\%$ of the entire vessel circumference (range 4–78 $\%$). The training set was generated with $\varOmega _1=10$ random pullbacks (corresponding to 82 images), and the testing set was generated from the remaining $\varOmega _2=21$ pullbacks (corresponding to 179 images).

**Fig. 4:**
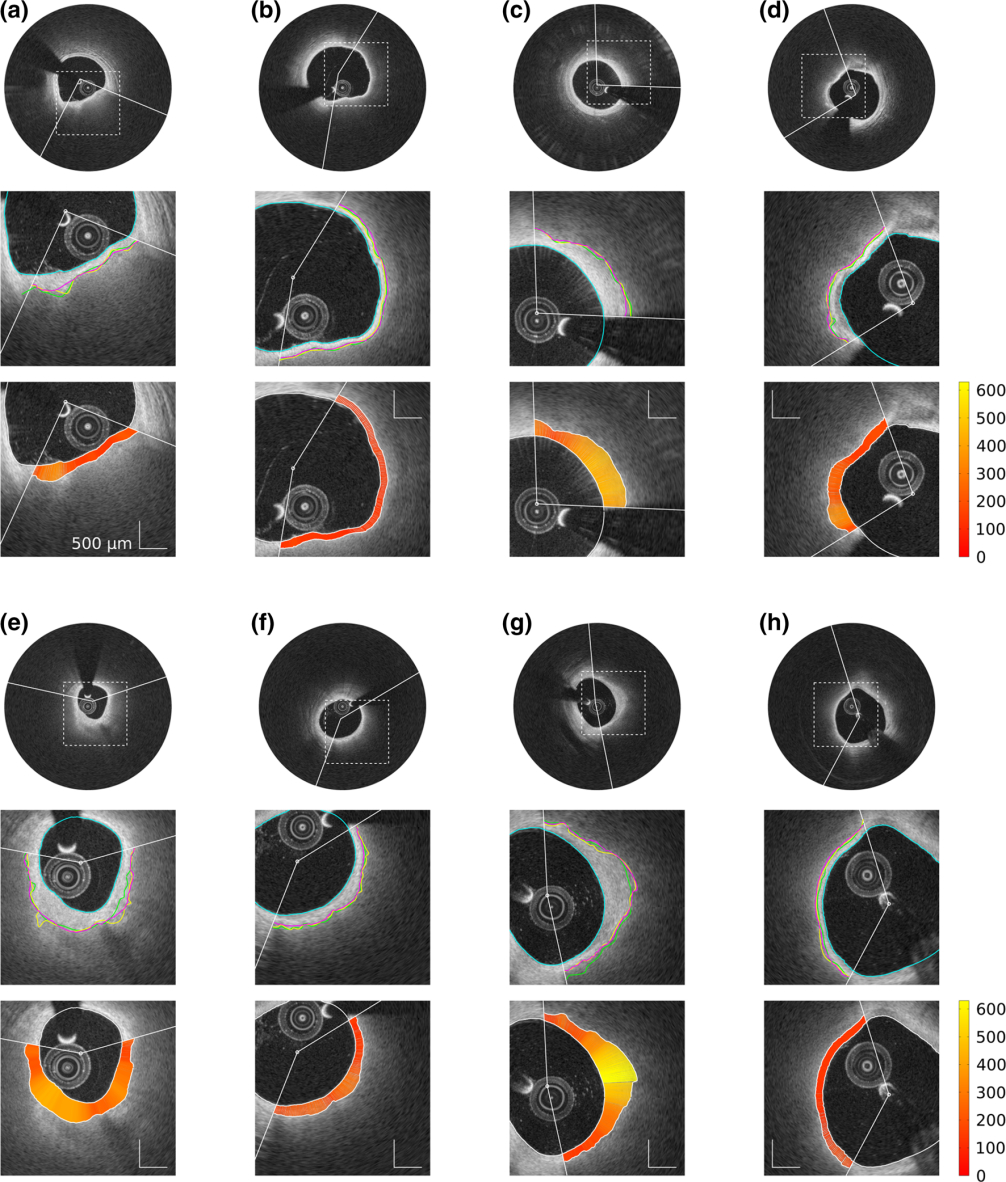
Representative results of the segmentation framework on eight frames from different pullbacks. For each example, the panel composition is the following. The *top row* displays the full image with the region of interest (ROI, *white arc*). The *middle row* displays an enlarged view of the region delimited by the *dashed square* in the *top row*. The automatic lumen segmentation is represented by the *cyan line*. Within the ROI, tracings of the abluminal interface performed by the segmentation method and the analysts ${\mathcal {A}}_1$ and ${\mathcal {A}}_2$ are represented by the *magenta*, *yellow*, and *green lines*, respectively. The *bottom row* displays the cap thickness (scale in $\upmu \hbox {m}$), automatically computed within the ROI as the distance between the luminal and abluminal contours that were extracted by the segmentation method

For each analyzed frame of both training and testing sets, the luminal interface was automatically extracted for the entire vessel circumference, and the abluminal interface of the fibrous cap was automatically extracted within the ROI defined by the expert ${\mathcal {A}}_1$ (Fig. 1a, b). Representative examples of resulting segmentation contours are displayed in Fig. 4. The results of our segmentation method, compared to the tracings of both observers ${\mathcal {A}}_1$ and ${\mathcal {A}}_2$, are presented alongside to the corresponding inter-observer variability in Table 2.

**Table 2:** Absolute segmentation errors (mean $\pm$ standard deviation) of the abluminal interface of the fibrous cap, for the automatic method (Auto) and the two analysts (${\mathcal {A}}_1$ and ${\mathcal {A}}_2$)

Errors $(\upmu \hbox {m})$	Auto versus ${\mathcal {A}}_1$	Auto versus ${\mathcal {A}}_2$	${\mathcal {A}}_1$ versus ${\mathcal {A}}_2$
Testing set $(\varOmega _2=21)$	31 $\pm$ 38	37 $\pm$ 41	30 $\pm$ 39
Training set $(\varOmega _1=10)$	31 $\pm$ 39	33 $\pm$ 33	34 $\pm$ 43

**Fig. 5:**
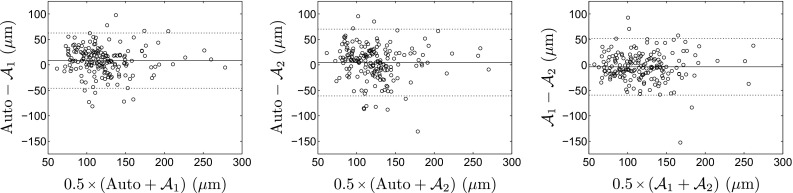
Bland-Altman plots, comparing the results of minimal cap thickness assessed in the training set, for the proposed automatic method and the manual tracings performed by the two analysts ${\mathcal {A}}_1$ and ${\mathcal {A}}_2$. The *solid* and *dashed lines* represent the bias and the $95\,\%$ limits of agreement, respectively

Quantification of fibrous cap thickness was derived from the segmented contours of both luminal and abluminal interfaces. Including each analyzed A-line per frame, the average cap thickness was $210 \pm 82~\upmu \text {m}$ for the 179 images of the testing set and $228 \pm 88~\upmu \text {m}$ for the 82 images of the training set. The mean minimal cap thickness (i.e., the thinnest point in a given frame) was $126 \pm 37~\upmu \text {m}$ for the testing set, and $161 \pm 64~\upmu \text {m}$ for the training set. Results of cap thickness derived from the automatic framework were evaluated against the manual references performed by the two analysts, as presented in Table 3. The Bland-Altman plots (Fig. 5) show an overall good agreement between the present method and the two experts when assessing minimal cap thickness.

**Table 3:** Evaluation of fibrous cap thickness, with absolute error (mean $\pm$ standard deviation), bias, $95\,\%$ limits of agreement (Lim), and Pearson coefficient (R), for the automatic method (Auto) and the two analysts (${\mathcal {A}}_1$ and ${\mathcal {A}}_2$)

Errors $(\upmu \hbox {m})$	Overall cap thickness over the entire ROI				Minimal cap thickness per frame			
	Absolute	Bias	Lim	R	Absolute	Bias	Lim	R
Testing set $(\varOmega _2=21)$								
Auto versus ${\mathcal {A}}_1$	30 $\pm$ 37	1.4	$[-92, 95]$	0.85	22 $\pm$ 18	8.4	$[-46, 63]$	0.73
Auto versus ${\mathcal {A}}_2$	36 $\pm$ 41	4.9	$[-101, 111]$	0.81	26 $\pm$ 22	4.6	$[-61, 70]$	0.62
${\mathcal {A}}_1$ versus ${\mathcal {A}}_2$	36 $\pm$ 41	3.6	$[-102, 109]$	0.82	21 $\pm$ 19	$-$3.8	$[-59, 52]$	0.74
Training set $(\varOmega _1=10)$								
Auto versus ${\mathcal {A}}_1$	31 $\pm$ 39	$-$1.4	$[-99, 96]$	0.86	30 $\pm$ 27	2.4	$[-77, 82]$	0.82
Auto versus ${\mathcal {A}}_2$	32 $\pm$ 33	$-$1.8	$[-91, 87]$	0.87	29 $\pm$ 27	2.0	$[-75, 79]$	0.85
${\mathcal {A}}_1$ versus ${\mathcal {A}}_2$	35 $\pm$ 41	$-$0.4	$[-105, 105]$	0.84	24 $\pm$ 25	$-$0.4	$[-68, 67]$	0.89

It is also insightful to quantify the absolute error of the proposed segmentation framework normalized by the cap thickness. Calculating, for each analyzed A-line, the ratio between the absolute segmentation error and the corresponding cap thickness and putting all these ratios together, the mean values were $16 \pm 19\,\%$ for the 179 images of the testing set and $15 \pm 22\,\%\,$ for the 82 images of the training set. When calculating the relative errors corresponding to the minimal cap thickness, the mean values were $19 \pm 18\,\%$ for the testing set and $24 \pm 31\,\%$ for the training set.

**Fig. 6:**
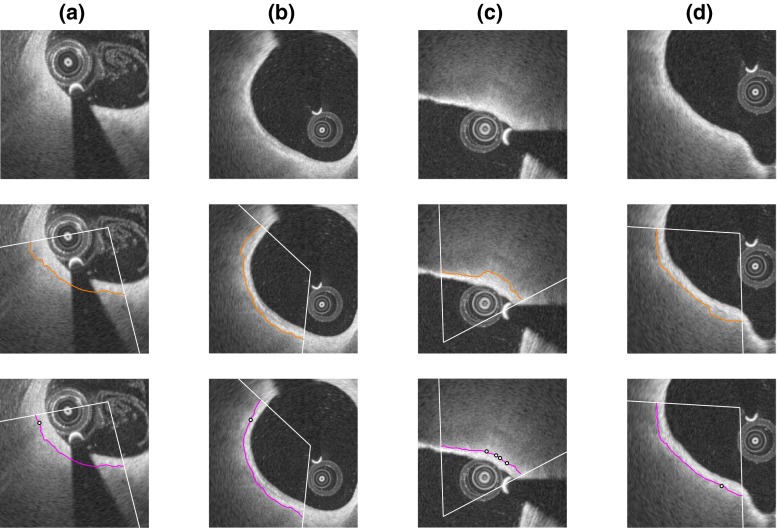
Example of manual contour correction, on four frames from different pullbacks. The *top*, *middle*, and *bottom rows* display the original image, the automatic segmentation contour of the fibrous cap (*orange line*), and the corrected segmentation contour (*magenta line*), respectively. In the *bottom row*, the control points that were manually indicated by the analyst are represented by the *black dots*

Reviewing the resulting abluminal interface segmentation contours of the testing set, the expert ${\mathcal {A}}_2$ performed a correction of the automatic contours with which he disagreed, as detailed in “Manual correction of the abluminal contour”. A total of 20 frames out of 179 were corrected, corresponding to seven pullbacks out of 21. For all these corrected frames, the mean number of manually added control points was $1.8 \pm 1.1$ (range 1–4). The two main factors motivating this manual corrections were (1) image artifacts hampering the automatic segmentation and (2) the presence of several interface-like structures attracting the contour. Examples of such manual correction of erroneous contours are depicted in Fig. 6. Assessing the fibrous cap with the corrected contours yielded an overall reduced cap thickness (bias of $-26~\upmu \text {m}$, Bland-Altman $95\,\%$ limits of agreement of $[-95, 147]~\upmu \text {m}$). Comparing, for the 20 corrected frames, the bias (and 95 % limits of agreement) of the cap thickness estimation resulting from the automatic segmentation and the manually corrected segmentation, it decreased from $22~\upmu \text {m}~([-142, 187]~\upmu \text {m})$ to $-4~\upmu \text {m}~([-125, 117]~\upmu \text {m})$ when evaluated against the reference tracings of ${\mathcal {A}}_2$, but increased from $1~\upmu \text {m}~ ([-133, 134]~\upmu \text {m})$ to $-26~\upmu \text {m}~([-159, 108]~\upmu \text {m})$ with ${\mathcal {A}}_1$. This discrepancy, reflecting the subjectivity of human analysts, is also visible through the bias between the two experts, which was equal to $21~\upmu \text {m}~([-124, 166]~\upmu \text {m})$ in these 20 frames.

As for the computational speed, the present framework required on average 0.5 s to perform the contour extraction of both luminal and abluminal interfaces and evaluate its thickness for a single image, while the corresponding manual operation required on average 190 s. In both cases and additionally, the average time (per frame) required by the user to define the ROI was 20 s.

## Discussion

The principal aim of this study was to introduce a contour segmentation method devised to quantify fibrous cap thickness in cross-sectional OCT images. Since cap thickness is the most critical component of plaque stability [11], its quantification is likely to provide crucial information about the risk of plaque rupture. The proposed framework was trained in 82 images from 10 patients and subsequently validated in 179 images from 21 other patients.

The evaluation of the proposed segmentation framework was conducted against reference segmentation contours manually generated by two expert analysts in a set of 179 images. The mean absolute error of the automatic method versus both analysts (i.e., $22\pm 18$ and $26\pm 22~\upmu \text {m}$) was similar to the inter-observer variability (i.e., $21\pm 19~\upmu \text {m}$), as presented in Table 3, which indicates that the present method performs at least as well as an experienced observer when assessing the cap thickness. It is also noteworthy that these errors are relatively small compared to the spatial resolution of the system, which was $20~\upmu \text {m}$. When assessing the thinnest portion of the fibrous cap in the image, an overall low positive bias was observed between the automatic method and the experts (i.e., $8.4~\upmu \text {m}$ w.r.t. ${\mathcal {A}}_1$, and $4.6~\upmu \text {m}$ w.r.t. ${\mathcal {A}}_2$), showing that cap thickness is slightly over-estimated by the computerized method. Furthermore, an accuracy improvement of roughly $30\,\%$ could be observed when quantifying, in a given frame, the thinnest portion of the cap rather than the overall thickness of the entire cap (i.e., $22\pm 18$ vs. $30\pm 37~\upmu \text {m}$, Table 3). This performance discrepancy can be explained by the fact that thinnest portions tend to present sharper and more defined contours, whereas thickest portions of the cap often present more fuzzy contours (i.e., due to a greater attenuation of the signal in deeper tissues or to a decrease in the lateral spatial definition along the distance from the probe in the Cartesian domain). We consider the higher accuracy in detection of minimal cap thickness favorable, as the minimal value of cap thickness is the most clinically relevant information [11]. It is also noteworthy that the ability of OCT to quantify cap thickness was previously evaluated in a study where cap thickness was measured manually in OCT images and compared to the corresponding ex vivo histopathologic segments [10]. Results from that study demonstrated a mean signed error of $-22\pm 44~\upmu \text {m}$ when measuring the thinnest portion of the cap. This magnitude can thus be understood as the systematic uncertainty that is introduced when analyzing cap thickness in OCT images. The level of accuracy of the proposed method can be validated by the fact that the mean signed error is small in comparison to that uncertainty, namely $-8.4\pm 28~\upmu \text {m}$ (Table 3).

A training phase was carried out on a subsample of $\varOmega _1=10$ pullbacks to determine the optimal set of the $\{\alpha , \beta , \sigma \}$ parameters for the extraction of the fibrous cap abluminal interface. Despite the fact that the method was optimized with respect to the manual tracings of the expert ${\mathcal {A}}_1$, results show that the errors of the automatic method versus both analysts were close to each other as well as to the inter-observer variability, for contour segmentation (Table 2) as well as cap thickness assessment (Table 3). Moreover, both automatic and manual errors generated from the training set were similar to the errors resulting from the testing phase (Tables 2, 3), which confirms the robustness of the proposed framework against new images. In a few challenging cases (i.e., 20 images out of 179), automatic segmentation of the abluminal interface failed, due to the presence of bright image artifacts or several interface-like structures. To cope with these issues, a correction scheme was proposed. This task is performed easily and quickly by the user visually assessing the resulting segmentation, and indicating, if necessary, one or several control points to modify the contour, as displayed in Fig. 6. As opposed to the abluminal contour, segmentation of the luminal interface does not present any particular challenge, as the location of the anatomical boundary is well perceptible (Fig. 1b). This is testified by the fact that the parameter settings used for the luminal segmentation assign less weight on the shape constraint and more weight on the image data (i.e., $\{\alpha ,\beta ,\sigma \}=\{0.1, 1.0, 90~\upmu \text {m}$}), compared to the abluminal parameter settings (i.e., $\{\alpha ,\beta ,\sigma \}=\{0.2, 1.8, 45~\upmu \text {m}$}).

The clinical context of our work relates to perioperative decision making rather than patient screening: the severity of the case is averred, and invasive imaging is required. The rationale of the present study is to assess plaque stability via quantifying the thickness of the overlying fibrous cap. Indeed, it has been demonstrated that cap thickness is the most critical component of plaque stability [11] and that lesion morphology is associated with future events [13]. The error introduced by the present framework when assessing minimal cap thickness corresponded to $22 \pm 18~\upmu \hbox {m}$. This is relatively large compared to the threshold of $65~\upmu \hbox {m}$ used to identify rupture-prone sites [17]. Nevertheless, the error of the automatic method was similar to the agreement between the two experts, which was $21 \pm 19~\upmu \hbox {m}$. One should also notice that the empirical $65~\upmu \hbox {m}$ threshold may be under-evaluated, since ex vivo tissues can undergo variable shrinkage rate during histological preparation [14, 17]. Indeed, it has recently been demonstrated that ruptured plaques in ACS are often associated with a fibrous cap thickness of up to $100~\upmu \hbox {m}$ [15] and that the best cutoff to predict rupture was $151~\upmu \hbox {m}$ for most representative fibrous caps [21]. Accordingly, the clinical applicability of the proposed method is supported by a relatively accurate quantification of cap thickness.

To the best of our knowledge, the study presented by Wang et al. [19] is the only one to report a semiautomatic segmentation scheme dedicated to quantify fibrous cap thickness in coronary OCT. The accuracy of that method was slightly better than that of the present framework, namely the mean absolute errors ($\pm$ standard deviation) were $25~(\pm 31)~\upmu \hbox {m}$ versus $31~(\pm 38)~\upmu \hbox {m}$ for the abluminal interface of the cap, and $27~(\pm 27)~\upmu \hbox {m}$ versus $30~(\pm 37)~\upmu \hbox {m}$ for the overall cap thickness. Nevertheless, the pertinence of such comparison is limited by the fact that our method was applied onto a different dataset, using a different OCT scanner, and that the protocol followed by the expert ${\mathcal {A}}_1$ to determine the fibrous caps to be analyzed may also have differed. Moreover, the finding of a higher inter-observer variability as well in our study could imply the presence of challenging cases in our dataset.

A limitation of this study is that the cap thickness validation was performed against tracings manually generated by expert analysts, but not against ex vivo histopathologic specimens. Therefore, the actual ground truth is lacking, and further validation is warranted. However, as a variable shrinkage rate often occurs during histological preparation of the tissues [14, 17], validation on ex vivo data is also expected to involve a certain amount of uncertainty. Another limitation of this study is that a manual initialization phase is required to be performed by the user to indicate the location of the ROI encompassing the fibrous cap to be analyzed. A certain amount of variability is to be expected in between two selections from the same experts, or in between the selection of two different experts, thus hindering clinical applicability. This could be remedied by a more automated way of detecting these locations. Therefore, future work will focus on fully automatic detection of such diseased regions, using an approach based on machine learning [22]. One should also notice that since the spatial resolution along the z-axis is rather coarse compared to the axial resolution (i.e., $200$ vs. $20~\upmu \text {m}$), a three-dimensional segmentation approach is not expected to greatly improve the overall accuracy. For this reason, the proposed framework is based on two-dimensional cross-sectional images. This issue could be addressed in further work by upsampling the acquired data using an ultrafast OCT system at 3200 frames per second [18]. To cope with the diffuse appearance of the abluminal contours, multiple texture features could also be extracted in addition to the intensity gradient in order to generate a multi-dimensional cost function ${\mathcal {C}}$. Future perspectives will also aim at investigating the association of wall shear stress with cap thickness using a fusion of imaging parameters with OCT and biplane angiography, in the objective to assess the risk of plaque rupture with improved performances. Potential applications could also include automated assessment of device-induced vascular responses [7, 8].

## Conclusion

The context of this study is to assess rupture-prone plaques by quantifying the thickness of the overlying fibrous cap in cross-sectional coronary OCT imaging. A segmentation framework devised to extract the contours of the cap was proposed. In the objective to localize the diffuse and fuzzy abluminal interface, the introduced method is based on a specific dynamic programming approach that integrates a geometrical a priori. Validated on in vivo data in 21 patients suffering from coronary artery disease, the method provided robust and accurate results, in a clinically acceptable computational time. The automatic framework performed as well as two expert analysts, while being substantially faster. Accordingly, the proposed approach could provide a useful aid for interventional planning and decision making in the catheterization laboratory.

## Footnotes

1 Please note that in the remaining of this manuscript, any reference to segmentation contour and all results are related to the original automatic contours (i.e., non manually corrected), except when explicitly specified.
